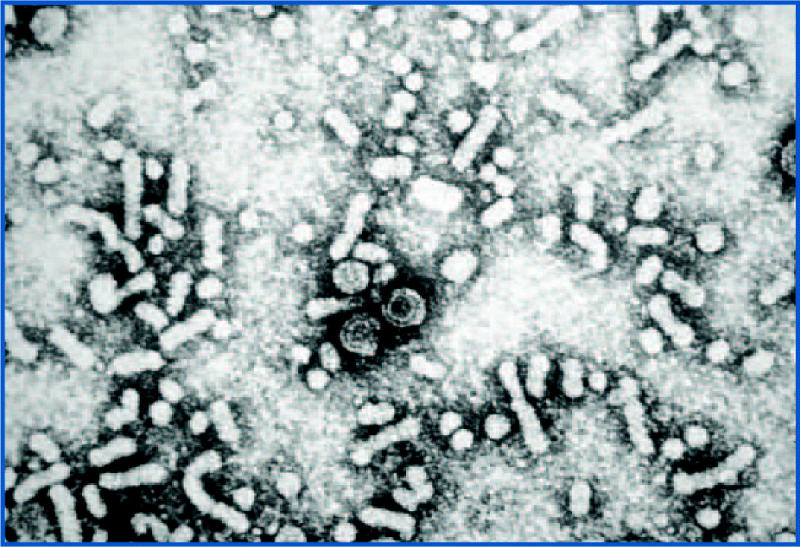# Headliners: Liver Cancer: Hepatitis B Virus Mutation Predicts Liver Cancer

**Published:** 2004-08

**Authors:** Jerry Phelps

Kuang SY, Jackson PE, Wang JB, Lu PX, Muñoz A, Qian GS, Kensler TW, Groopman JD. 2004. Specific mutations of hepatitis B virus in plasma predict liver cancer development. Proc Natl Acad Sci USA 101:3575–3580.

Liver cancer is the fifth most prevalent form of cancer worldwide, causing more than 500,000 deaths annually, according to the World Health Organization. Exposure to the hepatitis B virus (HBV) is a major risk factor in the development of liver cancer. Now a team including NIEHS grantees Alvaro Muñoz, John D. Groopman, and Thomas W. Kensler, all of the Johns Hopkins Bloomberg School of Public Health, has identified a biomarker that may predict future cases of liver cancer in HBV carriers.

Previous work by members of this team has shown that HBV exposure causes a 7-fold increase in risk. Exposure to aflatoxin, a mold product commonly found in peanuts and grains, increases the risk of liver cancer by 3.5 times. Combined exposure to these two agents results in a remarkable 60-fold increase in the risk of developing liver cancer. This is an especially troubling public health problem in China, where HBV and aflatoxin exposures are both very high.

In the current study, the researchers examined the prevalence a particular HBV mutation in the plasma and tumors of liver cancer patients living in Qidong, China. Initial studies determined that about three-fourths of the tumors from an initial group of 70 patients contained the mutation. In a second group of 15 liver cancer patients chosen from a cohort of high-risk individuals, the investigators determined that about half had detectable levels of the HBV mutation in their blood several years before the cancer appeared.

These findings suggest that detection of the mutated HBV in the blood is an early warning sign of subsequent liver cancer development, and suggest its use as an intermediate end point in prevention and intervention trials. **–Jerry Phelps**

## Figures and Tables

**Figure:**